# Effects of Inorganic Fluoride and the Fluoroquinolone Antibiotic Pefloxacin on the Growth and Microbiome Structure of *Eruca sativa* L.

**DOI:** 10.3390/ijms27072931

**Published:** 2026-03-24

**Authors:** Jan Kamiński, Agnieszka I. Piotrowicz-Cieślak

**Affiliations:** Department of Plant Physiology, Genetics and Biotechnology, University of Warmia and Mazury in Olsztyn, 10-720 Olsztyn, Poland

**Keywords:** phyllosphere, rhizosphere, microorganism

## Abstract

Environmental contamination with fluorinated compounds has increased markedly due to their widespread use in industry, medicine, and agriculture. Fluoride ions and fluoroquinolone antibiotics may enter soils through fertilizers, wastewater, and manure application, where they can interact with plant-associated microbial communities. In the present study, we investigated the effects of inorganic fluoride (applied as sodium fluoride, NaF) and the fluoroquinolone antibiotic pefloxacin on the growth and microbiome composition of *Eruca sativa* L. Plants were cultivated under controlled conditions and exposed for four weeks to NaF or pefloxacin at equimolar concentrations of 10 and 20 µM/kg soil. Morphological parameters, including biomass accumulation, root length, leaf dimensions, and leaf area, were not significantly affected by either treatment. Nevertheless, increased variability of growth traits was observed, particularly in plants exposed to NaF. High-throughput sequencing of the 16S rRNA gene revealed pronounced, treatment-specific alterations in both rhizosphere and phyllosphere bacterial communities. The rhizosphere microbiome was relatively stable at higher taxonomic levels but exhibited selective enrichment of Actinomycetota, including the class Thermoleophilia, under NaF exposure. In contrast, the phyllosphere microbiome showed strong sensitivity to fluoride, with a marked increase in Betaproteobacteria, dominated by Burkholderiales. Changes induced by pefloxacin were weaker and more diffuse. Our results demonstrate that plant-associated microbiomes respond to fluorinated compounds at concentrations that do not induce visible plant stress. The phyllosphere microbiome, in particular, represents a sensitive indicator of fluoride exposure and may serve as an early-warning system for environmental contamination.

## 1. Introduction

Plant-associated microbiomes form complex and dynamic assemblages of microorganisms that colonize roots, aerial tissues, and surrounding soil. These microbial communities play essential roles in plant nutrition, growth regulation, pathogen suppression, and adaptation to abiotic stress [1,2,3]. The rhizosphere microbiome is largely shaped by root exudates and soil physicochemical properties, whereas the phyllosphere represents a nutrient-limited and highly variable environment exposed to ultraviolet radiation, desiccation, and atmospheric deposition [4,5,6].

Anthropogenic contamination has emerged as a major driver of microbiome alteration in terrestrial ecosystems. Among environmental contaminants, antibiotics are of particular concern due to their persistence, biological activity at low concentrations, and ability to select for antibiotic-resistant bacteria [7,8,9]. Agricultural soils receive antibiotics through manure fertilization and irrigation with reclaimed water [10]. Numerous studies have demonstrated that antibiotic residues can alter soil microbial diversity, metabolic activity, and resistance gene pools, with potential consequences for ecosystem functioning and human health [11,12,13].

Fluoroquinolone antibiotics constitute a widely used class of synthetic antimicrobials characterized by the presence of a fluorine atom in their molecular structure. This substitution enhances antibacterial activity and stability but also contributes to environmental persistence [10,14]. Pefloxacin, a second-generation fluoroquinolone, is used in human medicine and has been detected in soils and sediments [15]. During environmental degradation processes, fluoroquinolones may release fluoride ions, introducing an additional stress factor for microbial communities [16].

Fluoride ions themselves are biologically active and can inhibit numerous enzymatic processes by forming complexes with metal cofactors or by interfering with phosphorylation reactions [17]. Although fluoride toxicity has been extensively studied in animals and humans, its effects on soil and plant-associated microbiomes remain poorly characterized. Importantly, fluoride may originate from multiple sources, including phosphate fertilizers, industrial emissions, and degradation of fluorinated organic compounds [18,19,20].

Leafy vegetables such as *Eruca sativa* L. (arugula) are particularly relevant models for studying microbiome responses to environmental contamination due to their short cultivation cycle and raw consumption. The microbiome of *E. sativa* has been shown to harbor diverse bacterial taxa, including potential human pathogens and antibiotic-resistant bacteria [21,22,23]. Alterations in the phyllosphere microbiome of edible plants may therefore have direct implications for food safety.

The objective of this study was to compare the effects of inorganic fluoride and an organic fluorine-containing antibiotic on the rhizosphere and phyllosphere microbiome of *E. sativa*. By applying equimolar concentrations of NaF and pefloxacin, we aimed to distinguish fluoride-specific effects from those associated with antibiotic activity and molecular structure.

## 2. Results

### 2.1. Morphological Response of Plants to Long-Term Soil Contamination

Four-week cultivation of *Eruca sativa* in uncontaminated horticultural soil resulted in homogeneous plant material with low variability of the analyzed morphological parameters. After this period, the mean fresh biomass was 1.0 ± 0.5 g, dry biomass approximately 0.10 ± 0.05 g, leaf length 6.0 ± 0.5 cm, root length 6.2 ± 1.0 cm, and leaf area approximately 5.0 ± 0.8 cm^2^. The low variance of these parameters indicates stable growth conditions and the absence of environmental stress during the initial phase of the experiment.

**Figure 1:**
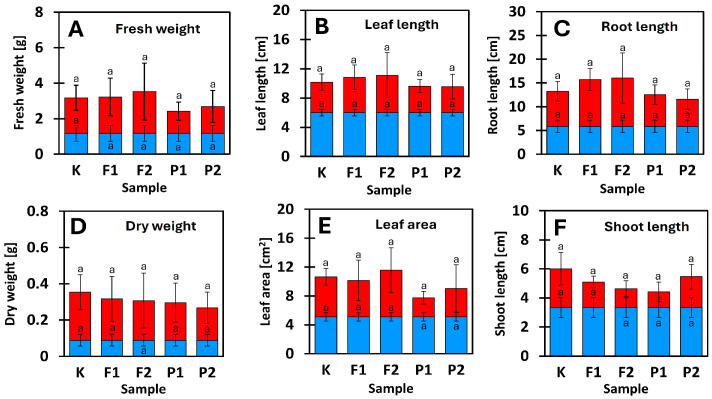
Morphology of *Eruca sativa* L. after 28 days—■, and 56 days—■ of growth. K—control (no fluoride addition); F1—sodium fluoride (NaF) at 10 µM/kg soil; F2—sodium fluoride (NaF) at 20 µM/kg soil; P1—pefloxacin at 10 µM/kg soil; P2—pefloxacin at 20 µM/kg soil. Panel (**A**) shows the mean fresh weight, panel (**B**) the mean leaf length, panel (**C**) the mean root length, panel (**D**) the dry weight, panel (**E**) leaf area, and panel (**F**) the mean shoot length of *E. sativa*. Data are presented as mean ± SD. Letters indicate statistically significant differences between groups (ANOVA, *p* < 0.05).

After an additional four weeks of growth in control soil and soils contaminated with NaF and pefloxacin at concentrations of 10 and 20 µM/kg soil, no statistically significant differences were observed between the mean values of the analyzed morphological parameters (ANOVA, *p* > 0.05). Fresh biomass increased to a range of 2.0–5.0 g, while dry biomass ranged from 0.20 to 0.50 g (Figure 1A,D). Despite the lack of differences in mean values, a marked increase in variability was observed in contaminated soils, particularly in the NaF 20 µM/kg treatment, where the coefficient of variation for fresh biomass exceeded 35%, compared to approximately 15% in the control.

A similar pattern was observed for dimensional parameters. Leaf length increased to 8–15 cm, root length to 10–22 cm, and leaf area to 6–15 cm^2^. The widest ranges of leaf length (8–15 cm) and root length (12–22 cm) were recorded in the NaF 20 µM/kg treatment, indicating increased inter-individual variability under fluoride exposure (Figure 1). Shoot length increased moderately from 3.5 ± 0.4 cm to 4–7 cm and was the only parameter for which variability remained comparable across all experimental variants.

### 2.2. Global Microbiome Restructuring in Response to Contamination

**Figure 2:**
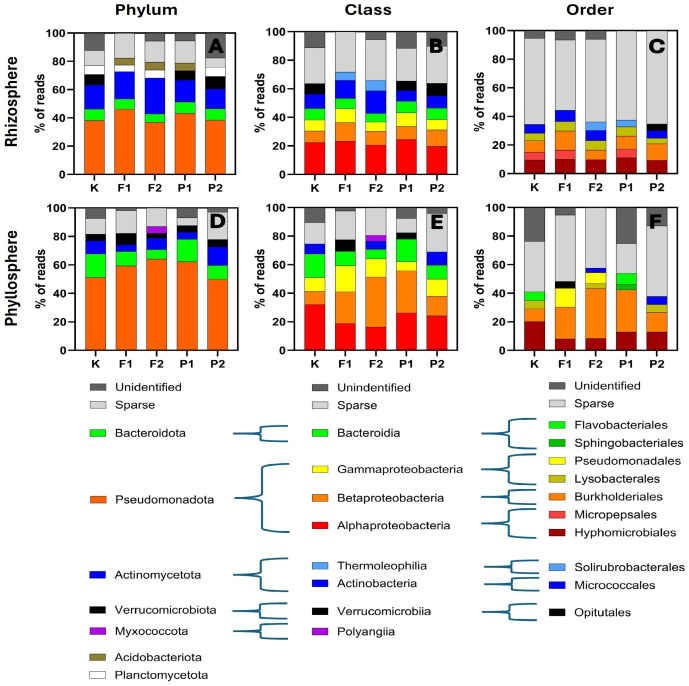
Relative abundance of amplicon sequence variants (ASVs) classified as unidentified (■), sparse (■), and assigned to major bacterial phyla (Bacteroidota (■), Pseudomonadota (■), Actinomycetota (■), Verrucomicrobiota (■), Myxococcota (■), Acidobacteriota (■), and Planctomycetota (□)), with further subdivision into classes and orders, in the rhizosphere and phyllosphere of *Eruca sativa.* Sample designations used in the figure: K—control without F-ions; F1—NaF at 10 µM/kg; F2—NaF at 20 µM/kg; P1—pefloxacin at 10 µM/kg; P2—pefloxacin at 20 µM/kg. ((**A**) Relative abundance of bacterial phyla in the rhizosphere; (**B**) relative abundance of bacterial classes in the rhizosphere; (**C**) relative abundance of bacterial orders in the rhizosphere; (**D**) relative abundance of bacterial phyla in the phyllosphere; (**E**) relative abundance of bacterial classes in the phyllosphere; (**F**) relative abundance of bacterial orders in the phyllosphere.

After 28 days of cultivation of *Eruca sativa* in soil supplemented with NaF and pefloxacin, the genomic structure of the rhizosphere and phyllosphere microbiomes was comprehensively characterized. Genomic DNA was isolated from soil and leaf samples collected from five experimental variants (control, 10 µM NaF, 20 µM NaF, 10 µM pefloxacin, 20 µM pefloxacin). Amplicon sequencing targeting the V3–V4 hypervariable region of the 16S rRNA gene was performed, and amplicon sequence variants (ASVs) were taxonomically assigned at the kingdom, phylum, class, order, family, genus, and species levels. Taxa representing less than 2% of ASVs within a given sample were grouped into a collective category designated as “Sparse.” Across all samples, both rhizospheric and phyllospheric, more than 99.8% of ASVs were assigned to Bacteria, with only a negligible fraction classified as Archaea (Supplementary Figure S1). This overwhelming bacterial dominance was consistent across all treatments. The genomic structure of both compartments comprised seven phyla: Bacteroidota, Pseudomonadota, Actinomycetota, Verrucomicrobiota, Myxococcota, Acidobacteriota, and Planctomycetota (Figure 2A,D).

In the rhizosphere, Bacteroidota, Acidobacteriota and Planctomycetota displayed relatively uniform abundances of approximately 6%, with variation not exceeding two percentage points between treatments. Actinomycetota accounted for approximately 12% in the control and both pefloxacin treatments, whereas their abundance increased markedly under NaF exposure, reaching 18% at 10 µM and 30% at 20 µM. Pseudomonadota constituted approximately 38% in the control, 20 µM NaF, and 20 µM pefloxacin, and approximately 47% in 10 µM NaF and 10 µM pefloxacin; these fluctuations did not show a clear dose-dependent relationship. Verrucomicrobiota were absent from NaF-treated samples. In the control group and at 10 µM pefloxacin, they accounted for approximately 7%, whereas at 20 µM they accounted for approximately 10%. Planctomycetota were not detected in 10 µM pefloxacin, while Acidobacteriota were absent in the control and 20 µM pefloxacin treatments (Figure 2A).

In contrast, the phyllosphere exhibited substantially greater variability (Figure 2D). Pseudomonadota increased progressively with rising NaF concentration, from 50% in the control to approximately 60% at 10 µM and 65% at 20 µM NaF. In 10 µM pefloxacin, their abundance resembled that observed in 20 µM NaF, whereas in 20 µM pefloxacin it approximated control levels. Conversely, Bacteroidota declined linearly with increasing NaF, decreasing from 15% in the control to 6% at 20 µM NaF. Their abundance in 10 µM pefloxacin was comparable to the control, and 9% in 20 µM pefloxacin. Actinomycetota and Verrucomicrobiota showed treatment-dependent changes without a consistent trend. Notably, Acidobacteriota and Planctomycetota were absent from the phyllosphere, whereas Myxococcota (6%) were detected exclusively in the 20 µM NaF treatment (Figure 2D). Class-level analysis refined the phylum-level observations (Figure 2B,E). In the rhizosphere, Bacteroidota were represented by Bacteroidia; Pseudomonadota by Alpha-, Beta-, and Gammaproteobacteria; Actinomycetota by Thermoleophilia and Actinobacteria; Verrucomicrobiota by Verrucomicrobiia; and Myxococcota by Polyangiia. No classes exceeding the Sparse threshold were identified within Acidobacteriota or Planctomycetota (Figure 2B). Verrucomicrobiia occurred in the same treatments as Verrucomicrobiota and at comparable abundances. Thermoleophilia were detected exclusively under NaF exposure (~5%). Bacteroidia were present in all samples (~5%). Gammaproteobacteria accounted for ~7% in the control and high-concentration treatments (20 µM NaF and 20 µM pefloxacin), and 10% at 10 µM of both compounds. Alphaproteobacteria remained stable at ~20% across all treatments. Betaproteobacteria constituted ~10%, with minor variation. Actinobacteria declined under pefloxacin (8%) relative to the control (10%) but increased in a NaF dose-dependent manner (13% and 16% at 10 and 20 µM NaF, respectively) (Figure 2B).

In the phyllosphere, all classes displayed pronounced treatment-dependent variation (Figure 2E). Betaproteobacteria increased dramatically with NaF concentration, from 8% in the control to 20% at 10 µM and 34% at 20 µM NaF. Under pefloxacin, they accounted for 28% (10 µM) and 12% (20 µM). Alphaproteobacteria decreased progressively with increasing NaF (32%, 18%, and 15%), while representing 25% and 23% in 10 and 20 µM pefloxacin, respectively. Gammaproteobacteria varied between 6% and 18% without a clear concentration-dependent pattern. Bacteroidia declined with increasing NaF concentrations, from 16% in the control to 10% and 5% at higher exposure levels. Thermoleophilia were not detected in the phyllosphere; however, Polyangiia (4%) were observed exclusively at 20 µM NaF. Verrucomicrobiia appeared in 10 µM NaF (8%) and 10 µM pefloxacin (4%). Actinobacteria were detected in the control (7%), 20 µM NaF (6%), and 20 µM pefloxacin (10%) (Figure 2E). At the order level, the contribution of the Sparse category and Unidentified ASVs increased substantially (Figure 2C,F). In the rhizosphere, Sparse represented approximately 60% of ASVs, while Unidentified did not exceed 6%. Opitutales, Solirubrobacterales, and Micropepsales each accounted for ~5% but occurred selectively across treatments. Hyphomicrobiales (~9%) and Lysobacterales (~6%, except 2% at 20 µM pefloxacin) were present in all samples. Burkholderiales varied between 5% and 13% without correlation to treatment. Micrococcales comprised ~5% in the control and 20 µM pefloxacin and ~8% in NaF-treated samples (Figure 2C).

In the phyllosphere, Sparse constituted ~36% in the control and NaF treatments, 20% in 10 µM pefloxacin, and 50% in 20 µM pefloxacin (Figure 2F). The proportion of Unidentified ASVs declined sharply with increasing NaF (24%, 5%, 0%), paralleling the increase in Burkholderiales, whose abundance closely mirrored that of Betaproteobacteria at the class level. Hyphomicrobiales represented 20% in the control, 8% in NaF treatments, and 14% in pefloxacin treatments. Micropepsales and Solirubrobacterales were absent from the phyllosphere. Pseudomonadales, Flavobacterales, and Sphingobacterales were detected but showed no consistent concentration-dependent patterns (Figure 2F).

### 2.3. Variability at the Family and Genus Levels

**Figure 3:**
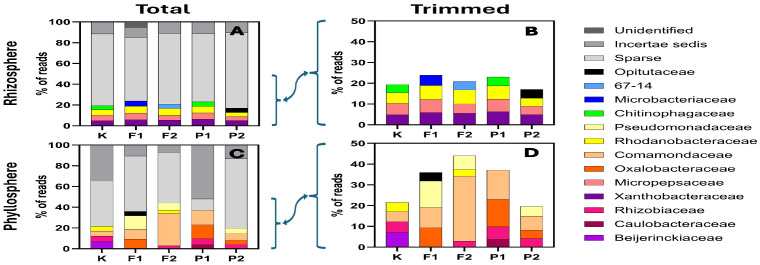
Percentage contribution of amplicon sequence variants (ASVs) assigned to unclassified (■), Incertae sedis (■), sparse (■), Opitutaceae (■), 67-14 (■), Microbacteriaceae (■), Chitinophagaceae (■), Pseudomonadaceae (■), Rhodanobacteraceae (■), Comamonadaceae (■), Oxalobacteraceae (■), Micropepsaceae (■), Xanthobacteraceae (■), Rhizobiaceae (■), Caulobacteraceae (■), and Beijerinckiaceae (■) families in the rhizosphere and the phyllosphere of *Eruca sativa*. Panel (**A**)—total rhizosphere reads; Panel (**B**)—rhizosphere reads rarefied to 50% of the total read count shown in Panel (**A**); Panel (**C**)—total phyllosphere reads; Panel (**D**)—phyllosphere reads rarefied to 50% of the total read count shown in Panel (**C**). Sample designations used in the figure: K—control without F-ions; F1—NaF at 10 µM/kg; F2—NaF at 20 µM/kg; P1—pefloxacin at 10 µM/kg; P2—pefloxacin at 20 µM/kg.

At the family level, Sparse increased to levels up to approximately 70% of ASVs, and Incertae sedis taxa appeared for the first time (Figure 3). In both compartments, the combined proportion of Sparse and Incertae sedis was at least 50%.

In the rhizosphere, Sparse exceeded 70% and Incertae sedis accounted for ~10% (Figure 3A,B). Identified families included Microbacteriaceae, 67-14 (an unnamed family within Solirubrobacterales), Chitinophagaceae, Rhodanobacteraceae, Micropepsaceae, and Xanthobacteraceae, each contributing approximately 5–7% with relatively consistent representation across treatments. Certain families were treatment-specific, such as Opitutaceae (20 µM pefloxacin), 67-14 (20 µM NaF), Microbacteriaceae (10 µM NaF), and Chitinophagaceae (control and 10 µM pefloxacin). In the phyllosphere, the combined proportion of Sparse and Incertae sedis ranged between 60% and 80%, with Incertae sedis varying markedly (7–50%) independently of treatment (Figure 3C). Identified families included Opitutaceae, Comamondaceae, Pseudomonadaceae, Rhodanobacteraceae, Oxalobacteraceae, Rhizobiaceae, Caulobacteraceae, and Beijerinckiaceae. Comamondaceae were present in all treatments and displayed a pattern closely paralleling that observed for Burkholderiales and Betaproteobacteria. Beijerinckiaceae were detected exclusively in the control (7%) (Figure 3C,D).

**Figure 4:**
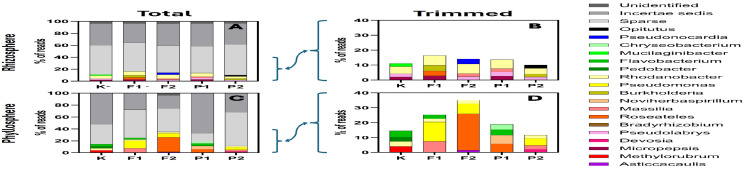
Percentage contribution of amplicon sequence variants (ASVs) assigned to unclassified (■), *Incertae sedis* (■), sparse (■), *Opitutus* (■), *Pseudomonocardia* (■), *Chryseobacterium* (■), *Mucilaginibacter* (■), *Flavobacterium* (■), *Pedobacter* (■), *Rhodanobacter* (■), *Pseudomonas* (■), *Burkholderia* (■), *Noviherbaspirillum* (■), *Massilia* (■), *Roseateles* (■), *Bradyrhizobium* (■), *Pseudolabrys* (■), *Devosia* (■), *Micropepsis* (■), *Methylorubrum* (■), and *Asticcacaulis* (■) genera in the rhizosphere and phyllosphere of *Eruca sativa*. Panel (**A**)—total rhizosphere reads; Panel (**B**)—rhizosphere reads rarefied to 40% of the total read count shown in Panel (**A**); Panel (**C**)—total phyllosphere reads; Panel (**D**)—phyllosphere reads rarefied to 40% of the total read count shown in Panel (**C**). Sample designations used in the figure: K—control without F-ions; F1—NaF at 10 µM/kg; F2—NaF at 20 µM/kg; P1—pefloxacin at 10 µM/kg; P2—pefloxacin at 20 µM/kg.

At the genus level, taxa other than Sparse and Incertae sedis accounted for less than 20% of ASVs in the rhizosphere and less than 40% in the phyllosphere (Figure 4). In the rhizosphere, Incertae sedis comprised ~37% and Sparse ~50%. Detected genera included Pseudonocardia, Mucilaginibacter (2% in control), Rhodanobacter, Opitutus, Burkholderia, Pseudolabrys, Roseateles, Micropepsis, and Massilia (Figure 4A,B).

In the phyllosphere, Mucilaginibacter, Micropepsis, Pseudonocardia, Burkholderia, and Opitutus were absent, whereas Pedobacter (3% in control), Flavobacterium, Chryseobacterium, Methylorubrum (4% in control), Asticcacaulis, Devosia, Pseudomonas, and Roseateles were identified (Figure 4C,D). Roseateles was particularly prominent at 20 µM NaF (25%), accounting for a substantial proportion of Comamondaceae (35%) in this treatment (Figure 3C,D and Figure 4C,D).

**Figure 5:**
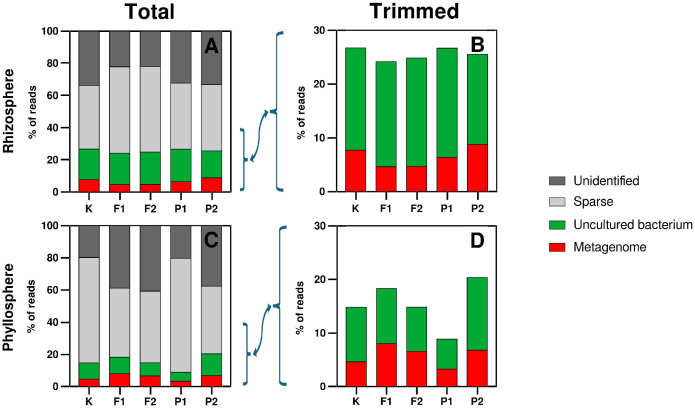
Percentage contribution of amplicon sequence variants (ASVs) assigned to unclassified (■), Sparse (■), Uncultured bacteria (■), Metagenome (■) genera in the rhizosphere and phyllosphere of *Eruca sativa*. Panel (**A**)—total rhizosphere reads; Panel (**B**)—rhizosphere reads rarefied to 30% of the total read count shown in Panel (**A**); Panel (**C**)—total phyllosphere reads; Panel (**D**)—phyllosphere reads rarefied to 30% of the total read count shown in Panel (**C**). Sample designations used in the figure: K—control without F-ions; F1—NaF at 10 µM/kg; F2—NaF at 20 µM/kg; P1—pefloxacin at 10 µM/kg; P2—pefloxacin at 20 µM/kg.

No species-level taxa exceeded the Sparse threshold applied during downstream filtering of low-abundance features (Figure 5). Amplicon sequence variants (ASVs) were inferred using the DADA2 algorithm, which models sequencing errors and does not rely on sequence similarity clustering. Taxonomic assignments were performed using a Naive Bayes classifier trained on the SILVA 138.2 database. In the rhizosphere, Sparse and Unidentified accounted for ~34% and ~40% in the control and pefloxacin treatments, whereas under NaF Unidentified declined to 22% and Sparse increased to 50%. Uncultured bacterium comprised approximately 20% across all treatments. The “Metagenome” category remained below 10% (7% in control, 5% under NaF, 6% and 9% in 10 and 20 µM pefloxacin, respectively) (Figure 5A,B).

In the phyllosphere, Unidentified represented 20% in the control and ~40% in NaF and 20 µM pefloxacin treatments. Sparse exhibited an inverse trend (60% in control and 10 µM pefloxacin; 40% in the remaining treatments). Uncultured bacterium accounted for 10% in the control and NaF treatments, 5% in 10 µM pefloxacin, and 15% in 20 µM pefloxacin. The “Metagenome” fraction increased under NaF exposure (4% in control to 7% and 6%) and measured 3% and 6% in 10 and 20 µM pefloxacin, respectively (Figure 5C,D). Collectively, these results demonstrate pronounced compartment-specific responses of the microbiome associated with *Eruca sativa*. The rhizosphere exhibited comparatively stable taxonomic composition with limited and non-linear treatment effects. In contrast, the phyllosphere displayed marked variability and clear NaF dose-dependent shifts, particularly within Betaproteobacteria, Burkholderiales, Comamondaceae, and the genus Roseateles (Figure 2, Figure 3 and Figure 4). These findings indicate distinct ecological dynamics and differential sensitivity of aboveground and belowground microbial communities to soil-applied chemical stressors.

### 2.4. Functional Analysis of the Genomes

In this study, microbial genes and metabolites were assigned to fifteen functional categories, including central carbon metabolism, carbohydrate metabolism, amino acid biosynthesis and degradation, lipid and fatty acid metabolism, nucleotide metabolism, vitamin and cofactor biosynthesis, cell wall and envelope biosynthesis, secondary metabolite biosynthesis, aromatic compound degradation, fermentation, nitrogen and sulfur metabolism, polyamine metabolism, and translation and protein biosynthesis. The functional categorization (Table S1) allows a structured overview of microbial metabolic capabilities, facilitates comparison across environmental conditions (e.g., fluoride and pefloxacin exposure), and provides a framework for interpreting metabolomic and genomic data in a biologically meaningful context.

**Figure 6:**
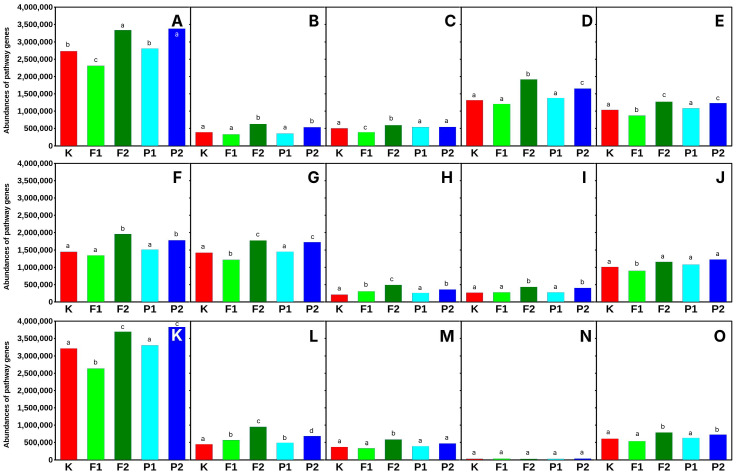
Functional categorization of microbial metabolic pathways in phyllosphere of *Eruca sativa*. of *Eruca sativa*. Fifteen biochemical pathways were analyzed, including: (**A**)—central carbon metabolism, (**B**)—carbohydrate metabolism, (**C**)—amino acid biosynthesis, (**D**)—amino acid degradation, (**E**)—lipid and fatty acid metabolism, (**F**)—nucleotide metabolism, (**G**)—vitamin and cofactor biosynthesis, (**H**)—cell wall and envelope biosynthesis, (**I**)—secondary metabolite biosynthesis, (**J**)—aromatic compound degradation, (**K**)—fermentation, (**L**)—nitrogen metabolism, (**M**)—sulfur metabolism, (**N**)—polyamine metabolism, and (**O**)—translation and protein biosynthesis. Sample designations used in the figure: K—control without F-ions; F1—NaF at 10 µM/kg; F2—NaF at 20 µM/kg; P1—pefloxacin at 10 µM/kg; P2—pefloxacin at 20 µM/kg. Different letters above bars indicate statistically significant differences between groups.

**Figure 7:**
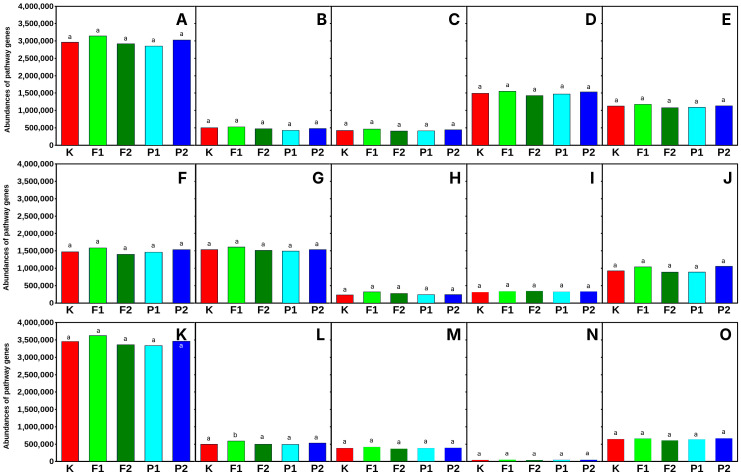
Functional categorization of microbial metabolic pathways in the rhizosphere of *Eruca sativa*. Fifteen biochemical pathways were analyzed, including: (**A**)—central carbon metabolism, (**B**)—carbohydrate metabolism, (**C**)—amino acid biosynthesis, (**D**)—amino acid degradation, (**E**)—lipid and fatty acid metabolism, (**F**)—nucleotide metabolism, (**G**)—vitamin and cofactor biosynthesis, (**H**)—cell wall and envelope biosynthesis, (**I**)—secondary metabolite biosynthesis, (**J**)—aromatic compound degradation, (**K**)—fermentation, (**L**)—nitrogen metabolism, (**M**)—sulfur metabolism, (**N**)—polyamine metabolism, and (**O**)—translation and protein biosynthesis. Sample designations used in the figure: K—control without F-ions; F1—NaF at 10 µM/kg; F2—NaF at 20 µM/kg; P1—pefloxacin at 10 µM/kg; P2—pefloxacin at 20 µM/kg. Different letters above bars indicate statistically significant differences between groups.

Significant functional changes in microbial metabolic pathways in response to fluoride (NaF) and pefloxacin (both at 20 µM/kg soil) were primarily observed in the phyllosphere (Figure 6) whereas the rhizosphere (Figure 7) remained largely stable under the same conditions. Among the analyzed pathways, the highest number of gene-level alterations occurred in nucleotide metabolism, central carbon metabolism, amino acid degradation, and vitamin and cofactor biosynthesis, indicating that these functional categories are particularly sensitive to chemical stressors at the higher exposure levels. In contrast, exposure to NaF at 10 µM/kg soil resulted in markedly fewer gene-level changes, with alterations in all pathways being even less than those observed in the untreated control, suggesting a threshold effect for fluoride-induced metabolic perturbations.

Overall, these findings indicate that microorganisms in the rhizosphere undergo dose-dependent metabolic adjustments in response to fluoride and pefloxacin, with high concentrations triggering substantial reprogramming in key functional categories. The phyllosphere-associated communities, however, remained relatively stable, highlighting the habitat-specific sensitivity of microbial metabolic networks. The differential responses of nucleotide metabolism, central carbon metabolism, amino acid degradation, and vitamin and cofactor biosynthesis underscore the importance of these pathways as metabolic hubs responsive to chemical stress in soil microbial communities.

### 2.5. Quantitative Summary and Biological Implications

The applied concentrations of NaF and pefloxacin (10 and 20 µM/kg soil) did not result in statistically significant changes in mean morphological parameters of *Eruca sativa* but led to a pronounced increase in inter-individual variability, reaching 20–30% compared to the control. At the same time, both contaminants induced substantial microbiome restructuring, with shifts in the relative abundance of dominant phyla and classes reaching 15–20 percentage points, particularly in the phyllosphere.

The rhizosphere exhibited relative quantitative and qualitative stability, whereas the phyllosphere responded rapidly and selectively, indicating its high sensitivity to sublethal chemical pressure. These results suggest that microbiome analysis, particularly of leaf-associated communities, may reveal environmental stress effects earlier than conventional plant growth parameters.

## 3. Discussion

The aim of the present study was to evaluate the effects of fluorine-containing compounds—sodium fluoride and the fluoroquinolone antibiotic pefloxacin—on the structure of the rhizosphere and phyllosphere microbiome of *Eruca sativa*, with particular emphasis on changes in the abundance and relative proportions of bacterial taxa. After four weeks of cultivation on contaminated soils, immediately prior to genomic DNA isolation, plant morphological parameters were assessed, including fresh and dry biomass, shoot and root length, and leaf length and width. One-way analysis of variance (*p* < 0.05) did not reveal statistically significant differences between treatments for any of the measured traits; however, a pronounced increase in result dispersion was observed in contaminated variants compared to the control (Figure 1). This indicates increased individual variability in plant responses under chemical stress. The applied concentrations of NaF and pefloxacin were below the levels that induce visible morphological changes in *E. sativa*, confirming that the observed microbial effects occurred under sub-toxic conditions for the host plant. The use of equimolar concentrations of NaF and pefloxacin allowed discrimination between effects attributable to fluoride ions and those potentially associated with the intact fluoroquinolone molecule. This approach is methodologically important, as previous studies have demonstrated that the fluorine moiety of fluoroquinolones may dissociate from the carbon skeleton and undergo independent environmental dispersion [16]. The obtained results clearly demonstrate that both compounds exert a significant influence on the plant-associated microbiome; however, the direction and magnitude of these effects depend on the ecological niche (rhizosphere vs. phyllosphere), contaminant concentration, and taxonomic resolution level (Figure 2). Taxonomic identification at the family and genus levels revealed a high contribution of taxa belonging to the “sparse” group, defined as accounting for less than 2% of total reads. This pattern indicates a high microbial diversity in both the rhizosphere and phyllosphere, which persisted even under NaF and pefloxacin exposure (Figure 3 and Figure 4). In the rhizosphere, taxa exceeding the sparse threshold collectively accounted for approximately 20% of all reads, whereas in the phyllosphere this proportion reached nearly 40%. This difference was largely attributable to the family Comamonadaceae within the order Burkholderiales (Figure 3C,D). At the order level, Burkholderiales accounted for approximately 40% of all phyllosphere reads (Figure 2F). The relative abundance of Comamonadaceae increased linearly with increasing NaF concentration. Although Comamonadaceae also increased in pefloxacin-treated samples, this effect was less pronounced than in fluoride-treated variants (Figure 3C,D). At the phylum levels, both rhizosphere and phyllosphere microbiomes were dominated by Pseudomonadota (ranging from approximately 40% to 60% of reads), Bacteroidota (~10–20%), Actinomycetota (~10–30%), Planctomycetota, and Verrucomicrobiota. This composition is consistent with previous reports on soil and plant-associated microbiomes, including those of leafy vegetables [24,25,26,27,28,29]. Despite the apparent stability of dominant phyla, the presence of NaF and pefloxacin resulted in substantial shifts in their relative proportions, highlighting the functional plasticity of microbial communities in response to chemical stress. In the rhizosphere, Verrucomicrobiota exhibited a particularly pronounced response. Their abundance dropped below Sparse threshold in the presence of NaF, whereas in pefloxacin-treated samples it remained comparable to the control at the lower concentration and increased at the higher concentration. This divergent response suggests that fluoride ions exert a stronger selective pressure on Verrucomicrobiota than fluorine bound within an organic antibiotic molecule. Similar patterns were reported by Jiang et al. [24], who observed an increase in Verrucomicrobiota at low fluoride concentrations and a decline at higher levels, interpreted as exceeding a tolerance threshold. In the present study, the applied concentrations were considerably lower, which may partially explain the differences in response patterns observed among treatments. Planctomycetota decreased from approximately 6% in the control to 4% in the presence of NaF, were not detected at 10 μM pefloxacin, and remained unaffected at 20 μM pefloxacin. Planctomycetota are considered relatively resistant to environmental stressors, including heavy metals and inorganic pollutants, which may explain their ecological success under fluoride exposure [30,31]. The absence of a response to pefloxacin suggests that the antibiotic exerts a more selective influence on the rhizosphere microbiome rather than a broad-spectrum effect across dominant bacterial groups. Phyllosphere-derived samples displayed a distinctly different response pattern, emphasizing the importance of plant microhabitats in shaping microbial community structure. A notable observation was the decline of Actinomycetota at lower NaF concentrations followed by a marked increase at higher concentrations; a similar non-linear response was observed in pefloxacin-treated samples. Such concentration-dependent responses are well documented and are often interpreted as the result of niche replacement following the elimination of more sensitive taxa [24,25,26,27,28,29]. The increased abundance of Actinomycetota at higher contaminant levels may reflect their adaptive capacity or their ability to exploit resources released by dying microorganisms. One of the most striking changes at lower taxonomic levels was the substantial increase in Burkholderiales abundance in phyllosphere samples. In NaF-treated variants, this order accounted for up to 40–50% of total reads, while in pefloxacin-treated samples its abundance was lower but still markedly higher than in the control. In NaF 10 and 20 µM/kg variants, Comamonadaceae was the dominant family within Burkholderiales, reaching up to 30% of all reads. This family has been identified as a primary environmental reservoir of the trimethoprim resistance gene *dfrB* [32], suggesting that fluoride stress may promote the selection of bacteria involved in the dissemination of antibiotic resistance genes. Moreover, Burkholderiales include numerous opportunistic human pathogens capable of causing severe infections, particularly in immunocompromised individuals and patients with cystic fibrosis [33], which confers additional public health relevance to the observed shifts. In the rhizosphere, a notable phenomenon was the disappearance of Chitinophagaceae (Bacteroidota) in NaF (10 and 20 µM/kg soil) and pefloxacin (20 µM/kg soil) variants. This family plays an important role in plant growth promotion and nitrogen assimilation [34,35,36], and its elimination may indicate a weakening of beneficial plant–microbiome interactions under chemical stress. Similarly, Microbacteriaceae (Actinomycetota), which also contribute to plant growth promotion [37], were detected exclusively in the rhizosphere of the NaF 10 µM/kg variant, indicating high sensitivity to both fluoride ions and pefloxacin. Beijerinckiaceae and Caulobacteraceae represent an interesting example of copiotrophic bacteria capable of rapidly increasing in abundance under conditions of reduced microbial diversity [38,39]. In the present study, Beijerinckiaceae were detected exclusively in the control phyllosphere and declined to sparse levels in all contaminated variants. Caulobacteraceae exceeded the sparse threshold only in the phyllosphere under low pefloxacin concentration, suggesting colonization of niches vacated by antibiotic-sensitive taxa. Importantly, Caulobacteraceae are recognized as significant environmental carriers of antibiotic resistance genes, often linked to nitrogen metabolism pathways [40]. From the perspective of antibiotic resistance ecology, one of the most significant findings was the complete disappearance of the genus *Pedobacter* in all NaF- and pefloxacin-treated samples. In the control phyllosphere, *Pedobacter* accounted for approximately 2% of total reads, whereas in all contaminated variants it declined to sparse levels. *Pedobacter* species are considered “superbugs”, exhibiting resistance to multiple antibiotic classes, including aminoglycosides, β-lactams, colistin, and ciprofloxacin, with resistance genes constituting up to 6–8% of their genome [41]. The disappearance of *Pedobacter* in pefloxacin-treated samples suggests sensitivity to this fluoroquinolone despite its structural similarity to ciprofloxacin. The lack of prior reports on *Pedobacter* susceptibility to pefloxacin underscores the novelty and ecological relevance of this finding. At the genus level, several taxa with documented roles in plant growth promotion and nitrogen assimilation were identified, including *Mucilaginibacter* [42], *Rhodanobacter* [43], *Micropepsis* [44], *Methylorubrum* [45], *Asticcacaulis* [46], and *Devosia* [47]. Changes in their abundance between control and contaminated variants indicate that fluoride and antibiotic stress may modify the functional potential of the microbiome even without complete elimination of specific taxa. The increased abundance of some of these genera in NaF- and pefloxacin-treated samples may be attributed to the high nitrogen content of the horticultural soil and the capacity of these bacteria to rapidly exploit available resources.

Central carbon metabolism (Figure 6 and Figure 7) constitutes a fundamental metabolic network in microorganisms, integrating pathways responsible for energy production, redox balance, and the generation of biosynthetic precursors. Consequently, perturbations in these pathways may reflect metabolic adaptations of microorganisms to environmental stressors, including exposure to antimicrobial compounds. Fluoride is known to interfere with bacterial metabolism, particularly by targeting enzymes involved in glycolysis. One of the primary mechanisms of fluoride action is the inhibition of enolase, a key glycolytic enzyme responsible for the conversion of 2-phosphoglycerate to phosphoenolpyruvate. This inhibition reduces glycolytic flux and affects carbohydrate metabolism and ATP generation in bacterial cells [48,49]. Moreover, the reduction in phosphoenolpyruvate availability may disrupt the phosphotransferase system responsible for carbohydrate transport, thereby affecting both glucose uptake and central carbon metabolism [50]. Antibiotics may also influence central carbon metabolism in microorganisms. Increasing evidence suggests that antibiotic exposure induces metabolic reprogramming, particularly affecting pathways associated with energy production and respiration. For example, bactericidal antibiotics have been shown to stimulate metabolic activity and alter carbon flux through central metabolic pathways such as glycolysis and the tricarboxylic acid (TCA) cycle [51]. These metabolic changes may increase cellular respiration and promote the generation of reactive oxygen species, which contribute to antibiotic-induced bacterial cell death. Furthermore, antibiotic stress can lead to broader metabolic adjustments, including shifts in carbon utilization and alterations in metabolic intermediates linked to central carbon metabolism. Such metabolic responses may represent adaptive mechanisms that enable microorganisms to maintain energy balance and survive under antimicrobial pressure [52]. Taken together, these findings suggest that both fluoride and antibiotic exposure may significantly affect central carbon metabolism in microorganisms. As central carbon metabolism functions as a metabolic hub connecting numerous biochemical pathways (Table S3), its perturbation may lead to widespread metabolic reorganization and influence microbial adaptation to antimicrobial stress. Alterations in nucleotide metabolism suggest that fluoride and pefloxacin may interfere with DNA and RNA synthesis, potentially impacting replication and repair processes [52]. Similarly, changes in amino acid degradation could reflect shifts in protein turnover and nitrogen balance under stress conditions [53]. The observed effects on vitamin and cofactor biosynthesis indicate that essential coenzymes required for enzymatic activity may be limited, which could further constrain metabolic capacity [54].

Interestingly, the phyllosphere communities remained relatively stable, highlighting a habitat-specific sensitivity of microbial metabolism. These findings suggest that soil-associated microorganisms in the rhizosphere are more responsive to chemical stress, possibly due to higher exposure or metabolic flexibility required for survival in nutrient-rich and competitive environments.

### Study Limitations and Future Directions

A limitation of the present study is the lack of direct measurements of fluoride ion concentrations in soil and plant tissues during the experiment, which would allow a more precise assessment of exposure dynamics. In addition, the applied 16S rRNA gene metabarcoding approach does not provide information on the functional potential of the microbiome, including antibiotic resistance genes or metabolic pathways involved in fluoride tolerance. Future studies combining quantitative fluoride analyses with shotgun metagenomics or metatranscriptomics would enable a more comprehensive evaluation of the functional consequences of fluoride and antibiotic-driven microbial selection in the rhizosphere and phyllosphere.

## 4. Materials and Methods

### 4.1. Cultivation Conditions of Eruca sativa

*Eruca sativa* was cultivated in horticultural soil (see Table S2 for soil composition), with 120 g of soil placed in 8 × 8 × 8 cm pots. Two to three seeds were sown per pot. Plants were grown for 4 weeks at 25 °C under a photoperiod of 18 h light and 6 h darkness. After this period, basic plant morphological parameters were measured, and the soil was supplemented with either NaF (Chempur, Piekary Śląskie, Poland) or pefloxacin (mesylate salt; C_17_H_20_FN_3_O_3_, Merck, Darmstadt, Germany) at concentrations of 10 and 20 µM/kg of soil. Cultivation was then continued for an additional 4 weeks under the same conditions, after which plant morphological parameters were assessed again.

### 4.2. Measurement of Morphological Parameters

The following parameters were evaluated: fresh and dry biomass, root length, leaf length and width, and leaf surface area. Dry biomass was determined after drying cleaned plant material at 40 °C for 24 h. Leaf surface area was measured using ImageJ software v.1.54m, while the remaining measurements were performed using millimeter paper. Differences between experimental groups were analyzed using one-way analysis of variance (ANOVA) with a significance level of α = 0.05.

### 4.3. Isolation of Genomic DNA

#### 4.3.1. Rhizosphere

Bacterial genomic DNA from the rhizosphere of *E. sativa* was isolated using the DNeasy PowerSoil Pro Kit (Qiagen, Hilden, Germany), according to the manufacturer’s instructions.

#### 4.3.2. Phyllosphere

Bacteria from *E. sativa* leaf tissues were isolated according to the protocol described by Krupka and Piotrowicz-Cieślak (2024), with minor modifications [55]. Leaves (5 g) were placed in 50 mL of sterile phosphate buffer (0.01 M, pH 7.4), subjected to sonication for 7 min, and subsequently shaken for 1 h at 180 rpm and 30 °C. The suspension was filtered through sterile gauze, and bacteria were collected on a PVDF membrane with a pore size of 0.22 µm. The membranes were stored at −80 °C prior to DNA isolation, which was also performed using the DNeasy PowerSoil Pro Kit.

#### 4.3.3. Microbiome Metabarcoding

DNA sequencing and taxonomic identification of bacteria from the rhizosphere and phyllosphere were outsourced to Genomed S.A. (Warsaw, Poland). DNA libraries were prepared by amplification of the hypervariable V3–V4 regions of the 16S rRNA gene, and sequencing was performed on the MiSeq platform (Illumina, San Diego, CA, USA) using paired-end reads method. Bacterial taxa were identified based on sequence comparison of the V3–V4 regions.

#### 4.3.4. Library Preparation

Microbial DNA isolated from phyllosphere and rhizosphere of *Eruca sativa* was used for preparation of DNA libraries for metabarcoding analysis. Libraries were generated using a two-step PCR protocol targeting the V3–V4 hypervariable region of the bacterial 16S rRNA gene. In the first PCR reaction, the V3–V4 fragment was amplified using locus-specific primers containing Illumina overhang adapters. The primers used were:

Forward primer (5′ → 3′): CCTACGGGNGGCWGCAG
Reverse primer (5′ → 3′): GACTACHVGGGTATCTAATCC
PCR amplification conditions for the first stage are presented in Table S3. In the second PCR step, unique dual indices and sequencing adapters were attached to the amplicons using index primers (Table S4).

#### 4.3.5. NGS Sequencing

Indexed libraries containing amplified V3–V4 fragments of the 16S rRNA gene were subjected to next-generation sequencing using a paired-end sequencing approach on an AVITI™ platform (Element Biosciences, San Diego, CA, USA).

#### 4.3.6. Bioinformatical Analysis

Raw sequencing reads were demultiplexed and converted to FASTQ format using the MiSeq Reporter v2.6 software. Downstream bioinformatic analysis was performed using QIIME2 (version 2024.5) [56].

Primer and adapter sequences were removed using Cutadapt (version 4.7) [57]. Quality filtering was applied to remove low-quality reads (Phred score < 30) and very short sequences.

Denoising, error correction, and inference of amplicon sequence variants (ASVs) were performed using the DADA2 algorithm [58] implemented within QIIME2. During this step, paired-end reads were merged into full-length V3–V4 sequences, and reads that could not be successfully merged were discarded. Chimeric sequences were identified and removed as part of the DADA2 pipeline.

Taxonomic classification of the resulting ASVs was performed using the SILVA reference database (release 138.2) [59]. A hybrid classification strategy was applied:

1. Initial taxonomic assignment using VSEARCH 2.30.0 [60] based on sequence similarity against the reference database.
2. ASVs that could not be confidently classified in the first step were further assigned using a Naive Bayes classifier trained on the SILVA 138.2 database with a minimum confidence threshold of 0.7.

Multiple sequence alignment of representative ASVs was performed using MAFFT [61]. A phylogenetic tree was subsequently constructed using FastTree [62]. Both tools are implemented within the QIIME2 framework.

#### 4.3.7. Functional Profiling of ASV Data

Functional potential of microbial communities was inferred using PICRUSt2, implemented through the q2-picrust2 plugin within the QIIME2 environment. This approach was used to predict the abundance of metabolic pathways based on the identified ASVs.

### 4.4. Statistical Analyses

All experiments were conducted with five biological replicates. One-way ANOVA with Tukey’s post hoc test was used to assess the differences in DNA concentration between extraction methods. Statistical analyses were performed using GraphPad Prism 8.0.1.

## 5. Conclusions

In summary, the results of this study demonstrate that both fluoride ions and pefloxacin exert strong selective pressure on the microbiome of *Eruca sativa*, leading to substantial alterations in the structure of rhizosphere and phyllosphere bacterial communities. These changes include the elimination of beneficial plant-associated taxa, selection of potentially resistant bacteria, and shifts among groups of ecological and health relevance. The findings contribute to a deeper understanding of the mechanisms by which fluorinated compounds influence environmental microbiomes and underscore the necessity of incorporating microbiome-level processes into environmental risk assessments related to antibiotic use. The results indicate that carbon metabolism was the most strongly affected metabolic category under the studied conditions. The highest number of alterations was observed in pathways associated with carbon processing, suggesting substantial changes in central metabolic functions. These findings highlight the key role of carbon metabolism in the organism’s response and suggest that shifts in carbon-related pathways may be an important component of the overall metabolic adaptation.

## Data Availability

The original contributions presented in this study are included in the article/Supplementary Material. Further inquiries can be directed to the corresponding author.

## Supplementary Materials

The following supporting information can be downloaded at: https://www.mdpi.com/article/10.3390/ijms27072931/s1.
