# Supplementary material for: Effects of Inorganic Fluoride and the Fluoroquinolone Antibiotic Pefloxacin on the Growth and Microbiome Structure of Eruca sativa L
Source: Int J Mol Sci. 2026 Mar 24;27(7):2931. doi: 10.3390/ijms27072931 (PMC13073882; doi:10.3390/ijms27072931)
Supplement: Supplementary file 1 [file ijms-27-02931-s001.zip › ijms-4193635-supplementary.pdf]

Effects of Inorganic Fluoride and the Fluoroquinolone Antibiotic Pefloxacin on the Growth and Microbiome Structure of *Eruca sativa* L.

Supplementary material

Jan Kamiński and Agnieszka I. Piotrowicz-Cieślak

Department of Plant Physiology, Genetics and Biotechnology, University of Warmia and Mazury in Olsztyn, 10-720 Olsztyn, Poland

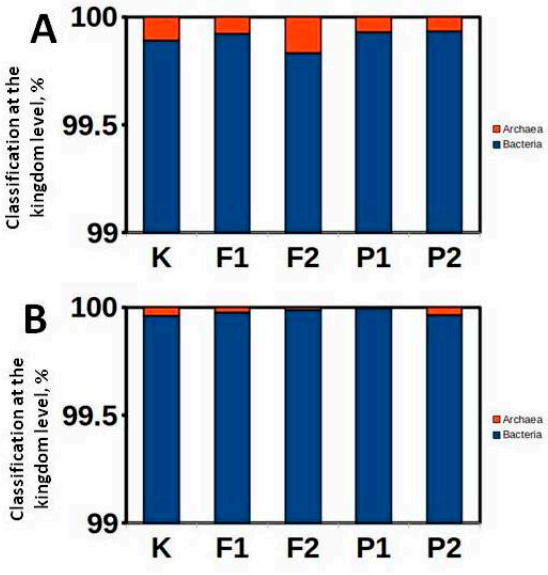

Figure S1. Isolated organisms (%) from the kingdoms Bacteria (■) and Archaea (■). Panel A: rhizosphere; Panel B: phyllosphere. Sample designations used in the figure: K – control without F^- ions; F1 – NaF at 10 μM/kg; F2 – NaF at 20 μM/kg; P1 – pefloxacin at 10 μM/kg; P2 – pefloxacin at 20 μM/kg.

Table S1. Functional categorization of predicted microbial metabolic pathways inferred from 16S rRNA gene data. Individual pathways (MetaCyc identifiers) were grouped into major metabolic categories (A–O) representing core cellular functions.

	**A.Central carbon metabolism**	
GLYCOLYSIS	GLYCOLYSIS-E-D	GLUCONEO-PWY
TCA	REDCITCYC	GLYOXYLATE-BYPASS
TCA-GLYOX-BYPASS	GLYCOLYSIS-TCA-GLYOX-BYPASS	PENTOSE-P-PWY
ANAGLYCOLYSIS-PWY		
	**B.Carbohydrate metabolism**	
FUCCAT-PWY	FUC-RHAMCAT-PWY	RHAMCAT-PWY
LACTOSECAT-PWY	GALACTUROCAT-PWY	GALACTARDEG-PWY
GALACT-GLUCUROCAT-PWY	GLUCARDEG-PWY	GLUCUROCAT-PWY
HEXITOLDEGSUPER-		

PWY		
**C. Fermentation**		
FERMENTATION-PWY	CENTFERM-PWY	ANAEROFRUCAT-PWY
**D. Lipid and fatty acid metabolism**		
FAO-PWY	FASYN-INITIAL-PWY	FASYN-ELONG-PWY
LIPASYN-PWY	PHOSLIPSYN-PWY	
**E. Cell wall and envelope biosynthesis**		
PEPTIDOGLYCANSYN-PWY	TEICHOICACID-PWY	LPSSYN-PWY
OANTIGEN-PWY	KDO-NAGLIPASYN-PWY	NAGLIPASYN-PWY
UDPNAGSYN-PWY		
**F. Vitamin and cofactor biosynthesis**		
BIOTIN-BIOSYNTHESIS-PWY	COBALSYN-PWY	FOLSYN-PWY
NAD-BIOSYNTHESIS-II	NADSYN-PWY	THISYN-PWY
PYRIDOXSYN-PWY	PANTO-PWY	PANTOSYN-PWY
**G. Secondary metabolite biosynthesis**		
ARO-PWY	COMPLETE-ARO-PWY	ALL-CHORISMATE-PWY
UBISYN-PWY	NONMEVIPP-PWY	POLYISOPRENSYN-PWY
**H. Aromatic compound degradation**		
CATECHOL-ORTHO-CLEAVAGE-PWY	PROTOCATECHUATE-ORTHO-CLEAVAGE-PWY	GALLATE-DEGRADATION-I-PWY
GALLATE-DEGRADATION-II-PWY	METHYLGALLATE-DEGRADATION-PWY	3-HYDROXYPHENYLACETATE-DEGRADATION-PWY
**I. Polyamine metabolism**		
POLYAMINSYN3-PWY	POLYAMSYN-PWY	ARG+POLYAMINE-SYN
**J. Nucleotide metabolism**		
DENOVOPURINE2-PWY	PRPP-PWY	SALVADEHYPOX-PWY
PYRIDNUCSYN-PWY	PYRIDNUCSAL-PWY	
**K. Amino acid biosynthesis**		
ARGSYN-PWY	ARGSYNBSUB-PWY	ASPASN-PWY
HISTSYN-PWY	TRPSYN-PWY	THRESYN-PWY
VALSYN-PWY	ILEUSYN-PWY	DAPLYSINESYN-PWY
BRANCHED-CHAIN-AA-SYN-PWY		
**L. Amino acid degradation**		
ARGDEG-PWY	ORNARGDEG-PWY	ORNDEG-PWY
HISDEG-PWY	LEU-DEG2-PWY	VALDEG-PWY
TYRFUMCAT-PWY		
**M. Translation and protein biosynthesis**		
TRNA-CHARGING-PWY	PPGPPMET-PWY	
**N. Nitrogen metabolism**		
DENITRIFICATION-PWY		
**O. Sulfur metabolism**		
SO4ASSIM-PWY	SULFATE-CYS-PWY	

Table S2. Quality specification of horticultural soil.

Parameter	Specification
Characteristic	Solid, loose form; fraction 0–25 mm; 100% high peat (degree of decomposition H3–H8); clay granulate; carbonate lime; organic NPK fertilizer with raw phosphate
pH (in H_2O)	5.5–6.5
Salt concentration	< 1.8 g NaCl/dm
Nitrogen (N)	200 mg/L
Phosphate (P_2O_5)	150 mg/L
Potassium oxide (K_2O)	200 mg/L
Magnesium (Mg)	120 mg/L
Sulfur (S)	120 mg/L

Table S3. Protocol of first stage of PCR reaction.

Cycle	Temperature	Duration	No. of cycles
Initial denaturation	98°C	30s	1
Denaturation	98°C	10s	25
Annealing	55°C	30s	
Elongation	72°C	20s	
Final elongation	72°C + 4°C	2min + hold	1

Table S4. Protocol of second stage of PCR reaction.

Cycle	Temperature	Duration	No. of cycles
Initial denaturation	98°C	30s	1
Denaturation	98°C	10s	7
Annealing	65°C	30s	
Elongation	72°C	20s	
Final elongation	72°C + 4°C	2min + hold	1
